# Adolescents’ Diabetes Self-Management Regimens and Outcomes During the COVID-19 Pandemic: A Scoping Review

**DOI:** 10.7759/cureus.76343

**Published:** 2024-12-24

**Authors:** Jason Nguyen, William Le, Roberta Brugger, Anjali Shah, Prasanna Karur, Macey Hedelund, John Joseph, Arshia Haj, Caroline Grillo, Nivene Hojeij, Jennifer Maizel

**Affiliations:** 1 Diabetes and Endocrinology, Nova Southeastern University Dr. Kiran C. Patel College of Osteopathic Medicine, Tampa, USA; 2 Diabetes and Endocrinology, Nova Southeastern University Dr. Kiran C. Patel College of Osteopathic Medicine, Fort Lauderdale, USA; 3 Public Health, Nova Southeastern University Dr. Kiran C. Patel College of Osteopathic Medicine, Fort Lauderdale, USA; 4 Behavioral Health and Health Policy Practice, Westat, Rockville, USA

**Keywords:** adherence, adolescence, continuous glucose monitoring, covid-19, diabetes, insulin, pandemic, telemedicine, weight

## Abstract

Adolescents with diabetes mellitus (DM) experience poorer glycemic outcomes and lower adherence to self-management regimens compared to other age groups. The coronavirus 2019 (COVID-19) pandemic posed new barriers to DM self-management, including social distancing measures and additional stressors. We conducted a scoping review of peer-reviewed literature to examine self-management regimens and outcomes among adolescents aged 10-17 years with type 1 and type 2 DM during the pandemic.

Our scoping review adhered to the Preferred Reporting Items for Systematic Reviews and Meta-Analyses Extension for Scoping Reviews (PRISMA-ScR) guidelines. We searched three online databases, screened articles through a rigorous process, and assessed bias using the Joanna Briggs Institute (JBI) Critical Appraisal Checklists. The findings from the included articles were categorized into six thematic areas: glycemic control/monitoring, insulin administration/regimens, weight/lifestyle behaviors, inpatient care/acute complications, outpatient care/telemedicine utilization, and psychosocial well-being.

The findings from the included articles (n = 32) varied. Adolescents who used continuous glucose monitoring (CGM), regularly adjusted insulin doses, and utilized telemedicine generally maintained or improved glycemic control during the pandemic. However, many adolescents gained weight, reduced their physical activities, worsened their diet and sleep habits, and experienced increased stress, all of which negatively impacted glycemic control. Rates of acute complications and hospitalizations varied among adolescents. Telemedicine was widely used and viewed positively by adolescents with DM.

Adolescents with DM faced various physical, behavioral, and psychosocial challenges during the COVID-19 pandemic. Further research is needed to assess the long-term impacts of the pandemic on this population. Multilevel interventions and preparedness efforts are required to improve and sustain adolescents’ DM self-management outcomes during public health emergencies, particularly focused on promoting CGM use, increasing physical activity levels, improving dietary habits, and reducing stress.

## Introduction and background

Diabetes mellitus (DM) is a chronic condition characterized by hyperglycemia, or high blood glucose levels [1]. It results when the pancreas fails to produce insulin (type 1) or when the body cannot effectively use insulin, known as insulin resistance (type 2) [1]. Hyperglycemia disrupts carbohydrate, lipid, and protein metabolism and, over time, can lead to a multitude of complications such as cardiovascular disease, retinopathy, neuropathy, diabetic kidney disease, and diabetic ketoacidosis (DKA) [1-5]. Globally, type 2 DM (T2DM) accounts for 90%-95% of diabetes cases, while T1DM accounts for 5%-10% [6]. However, T1DM is more prevalent than T2DM among adolescents [7]. In the United States, an estimated 352,000 individuals under 20 years of age were diagnosed with diabetes in 2021: 304,000 with T1DM and 48,000 with T2DM [7]. Notably, the prevalence of T2DM among adolescents is rising due to increasing rates of childhood obesity [7,8].

Both T1DM and T2DM are manageable conditions, with management primarily focused on achieving and maintaining glycemic control [9]. Ongoing management is essential for maintaining quality of life, reducing the risk of acute and long-term complications, and supporting mental health [9]. Regular monitoring of blood glucose levels is crucial for both T1DM and T2DM patients, either via continuous glucose monitoring (CGM) or capillary blood glucose monitoring (BGM) devices [10]. Therapies to maintain blood glucose levels include lifestyle modifications and pharmacological treatments [11-15]. Lifestyle modifications, such as healthy eating habits and regular physical activities, can sometimes eliminate the need for pharmacological treatments in individuals with T2DM [11,12]. The first-line pharmacological treatment for T1DM is insulin, administered via injections or a pump, while metformin is typically the first-line treatment for T2DM [6,13,14]. According to the updated guidelines from the American Diabetes Association (ADA) and the European Association for the Study of Diabetes (EASD), glycemic control is just one aspect of DM management [16]. Self-management education, regular medical appointments, and psychosocial support are also crucial for optimizing patient outcomes [9,17-19].

Ongoing management of T1DM and T2DM is especially critical for adolescents due to their age and increased lifetime risk of complications [20]. Research shows that adolescents have lower adherence to DM management regimens than other pediatric age groups and often fail to meet recommended glycemic targets, as measured by hemoglobin A1c (HbA1c) levels [20,21]. HbA1c reflects a patient’s average blood glucose level over a three-month period [6]. The ADA recommends that adolescents with T1DM and T2DM maintain HbA1c levels of <7% (53 mmol/mol); however, they emphasize that each patient’s HbA1c goal should be individualized and reassessed periodically [22]. Recent metrics from CGM, such as time in range (TIR), time below the target range, and time above the target range, provide more comprehensive insights into patients’ glycemic control over shorter periods, such as the past 14 days [23]. The ADA recommends that adolescents target a blood glucose range of 70-180 mg/dL with a high percentage of TIR [22].

Adolescents face physiological and psychosocial challenges that affect their glycemic control, including puberty, developmental behaviors, a growing sense of independence and identity, and exposure to new social situations and pressures [20]. They also report higher rates of mental health issues, such as depression, which can worsen diabetes management compared to younger pediatric age groups [20,24]. Furthermore, research shows that limited communication between parents and adolescents negatively impacts their diabetes management [25]. Adolescents tend to be more concerned about their self-image than other age groups, which can affect both glycemic control and psychological well-being [26].

It is estimated that less than 50% of individuals with DM adhere to their self-management regimens [27]. Patient-level barriers to adherence include limited knowledge of dietary guidelines and diabetes care plans, as well as feelings of helplessness and frustration due to poor glycemic control and disease progression [28,29]. Interpersonal barriers include limited social and familial support and communication challenges with healthcare providers, such as cultural and language differences [20,28-29]. Societal-level barriers include high costs of insulin, other medications, and supplies; limited access to healthcare and insurance; and diabetes-related stigma [28-30].

The COVID-19 pandemic posed new barriers to DM management [31]. For example, lockdowns and social distancing measures reduced patients’ access to routine diabetes care, medications, and supplies [31]. Furthermore, the pandemic caused financial hardships due to increased unemployment rates, and social distancing measures and school closures hindered patients’ ability to maintain physical activity levels, nutrition, and social support systems [31-33]. As a result, individuals with T1DM and T2DM experienced adverse health outcomes, such as hyperglycemia and increased body mass index (BMI) [34,35]. Research also indicates that people with diabetes face an increased risk of severe COVID-19-related illness, hospitalization, death, and mental health issues [35,36].

Given the recent studies highlighting barriers to DM self-management and the adverse impacts of the COVID-19 pandemic, a comprehensive review of the literature is necessary to assess changes in DM management and outcomes during the pandemic, as well as barriers to adherence. Research on the challenges faced by adolescents is crucial due to their unique physical and psychosocial vulnerabilities. To address this gap, we conducted a scoping review of peer-reviewed literature on T1DM and T2DM management among adolescents during the COVID-19 pandemic. This review examines adolescents’ glycemic control, blood glucose monitoring habits, medication regimens, weight management, lifestyle behaviors, hospitalizations, use of telemedicine and remote-delivered healthcare, acute diabetes complications, and psychosocial issues during the pandemic. Additionally, we discuss the implications of this literature for future research and improvements in public health and clinical practice. 

## Review

Materials and methods

We chose to conduct a scoping review because it is an effective first step in understanding the literature on a specific topic and provides a foundation for future systematic reviews and meta-analyses [37]. Our scoping review adhered to the PRISMA-ScR guidelines [38].

Search Strategy

We searched the following databases: Embase (Elsevier), Ovid MEDLINE (US National Library of Medicine), and Web of Science (Clarivate). These databases were selected for their broad international coverage of health research articles. Search strategies, including keywords and index terms, were adapted for each database. We applied search limiters such as peer-reviewed articles only, English language, publications since January 1, 2020, and participants between the ages of 7-12 and 13-17. Search terms and limiters are provided in Table 1 (Embase), Table 2 (Ovid MEDLINE), and Table 3 (Web of Science). All searches were completed on September 20, 2023.

**Table 1:** EMBASE Search Strategy Search Field Abbreviations: ab: abstract, ti: title, kw: author keywords, py: publication year, lim: limit to.

Line #	Search terms and limiters
1	(“type 1 diabetes” or “T1D” or “insulin dependent diabetes mellitus“ or “dm1” or “t1dm”):ab,ti,kw
2	(“type 2 diabetes” or “T2D” or “non insulin dependent diabetes mellitus” or “dm2” or “t2dm”):ab,ti,kw
3	Line #1 OR Line #2
4	(“COVID-19” or “COVID” or “Coronavirus” or “Pandemic”):ab,ti,kw
5	Line #3 AND Line #4
6	Line #5 AND (2020:py OR 2021:py OR 2022:py OR 2023:py) AND ([adolescent]/lim OR [school]/lim) AND [english]/lim

**Table 2:** Ovid MEDLINE Search Strategy Search Field Abbreviations: ab: abstract, ti: title, kw: keyword heading, yr: year of publication.

Line #	Search terms and limiters
1	(“type 1 diabetes” or “T1D” or “insulin dependent diabetes mellitus“ or “dm1” or “t1dm”):ab,ti,kw
2	(“type 2 diabetes” or “T2D” or “non insulin dependent diabetes mellitus” or “dm2” or “t2dm”):ab,ti,kw
3	Line #1 or Line #2
4	(“COVID-19” or “COVID” or “Coronavirus” or “Pandemic”):ab,ti,kw
5	Line #3 AND Line #4
6	limit 5 to (english language and full text and yr="2020 - 2023")

**Table 3:** Web of Science Search Strategy Search Field Abbreviations: TI: title, AB: abstract, KP: keyword plus.

Line #	Search terms and limiters
1	((TI=(“type 1 diabetes” or “T1D” or “insulin dependent diabetes mellitus“ or “dm1” or “t1dm”)) OR AB=(“type 1 diabetes” or “T1D” or “insulin dependent diabetes mellitus“ or “dm1” or “t1dm”)) OR KP=(“type 1 diabetes” or “T1D” or “insulin dependent diabetes mellitus“ or “dm1” or “t1dm”)
2	((TI=(“type 2 diabetes” or “T2D” or “non insulin dependent diabetes mellitus“ or “dm2” or “t2dm”)) OR AB=(“type 2 diabetes” or “T2D” or “ non insulin dependent diabetes mellitus“ or “dm2” or “t2dm”)) OR KP=(“type 2 diabetes” or “T2D” or “non insulin dependent diabetes mellitus“ or “dm2” or “t2dm”)
3	Line #1 OR Line #2
4	((TI=(“COVID-19” or “COVID” or “Coronavirus” or “Pandemic”)) AND AB=(“COVID-19” or “COVID” or “Coronavirus” or “Pandemic”)) AND KP=(“COVID-19” or “COVID” or “Coronavirus” or “Pandemic”)
5	Line #3 AND Line #4
6	Line #5 and 2020 or 2021 or 2022 or 2023 (Publication Years) and English (Languages)

Eligibility Inclusion/Exclusion Criteria

The inclusion criteria for our scoping review were as follows: original research articles published in peer-reviewed journals, studies addressing the management of T1DM and/or T2DM in adolescents, studies involving patients with a prior DM diagnosis, and articles with full texts that were either available or requestable. The review included studies enrolling adolescent participants aged 10-17 years [39]. For studies that included both the 10-17 age group and other age groups, only data specific to participants aged 10-17 years were reviewed if this information was clearly stated. We included studies employing various research designs (e.g., cohort, controlled, and qualitative studies) and temporal frameworks (e.g., cross-sectional and longitudinal data analyses) but excluded case studies. Studies focusing exclusively on newly diagnosed DM patients (e.g., within the past month) or systemic complications of DM were also excluded. Furthermore, dissertations, theses, conference abstracts, and book chapters were not considered.

Study Selection

Articles were screened using Rayyan (Rayyan, Cambridge, MA, United States) [40], an online research collaboration platform. Duplicates were first identified and removed. To ensure consistency in inclusion and exclusion decisions, nine authors conducted a calibration screening exercise. During calibration, each author reviewed the same 20% subset of article titles and abstracts. Interrater reliability (IRR) was assessed using Fleiss’ kappa [41], calculated in R® statistical software (R Foundation for Statistical Computing, Vienna, Austria), to determine the level of agreement. The IRR calculation indicated a strong agreement (k = 0.924). Following calibration, authors worked in pairs to screen the titles and abstracts of the remaining articles. Full-text screenings were also conducted by the same pairs. In cases of disagreement, a third author reviewed the article and made the final inclusion/exclusion decision.

Data Extraction and Synthesis

Two authors extracted data from the included articles using Microsoft Excel (Microsoft Corporation, Redmond, WA, United States). Extracted information included author(s), year of publication, study objective(s), study design, study setting, participants’ demographic characteristics, DM management measures, key findings, and study limitations. The synthesis focused on adolescents’ T1DM and T2DM management regimens during the COVID-19 pandemic, categorized into six thematic areas: (1) glycemic control and monitoring, (2) medication/insulin administration and regimens, (3) weight management and lifestyle behaviors, (4) acute complications and inpatient care, (5) outpatient care and telemedicine usage, and (6) psychosocial issues.

Risk of Bias Appraisals

Two authors independently assessed the articles for potential bias using the JBI Critical Appraisal Checklists [42]. These checklists evaluate various research biases, overall congruence, and essential methodological components. Articles were categorized based on their risk of bias: high (scores < 50%), moderate (scores between 50% and 70%), or low (scores > 70%) [42]. Each included study was evaluated using the checklist specific to its design. For instance, cross-sectional studies were assessed using the JBI Critical Appraisal Checklist for Analytical Cross-Sectional Studies.

Results

Article Selection and Critical Appraisal Results

**Figure 1:**
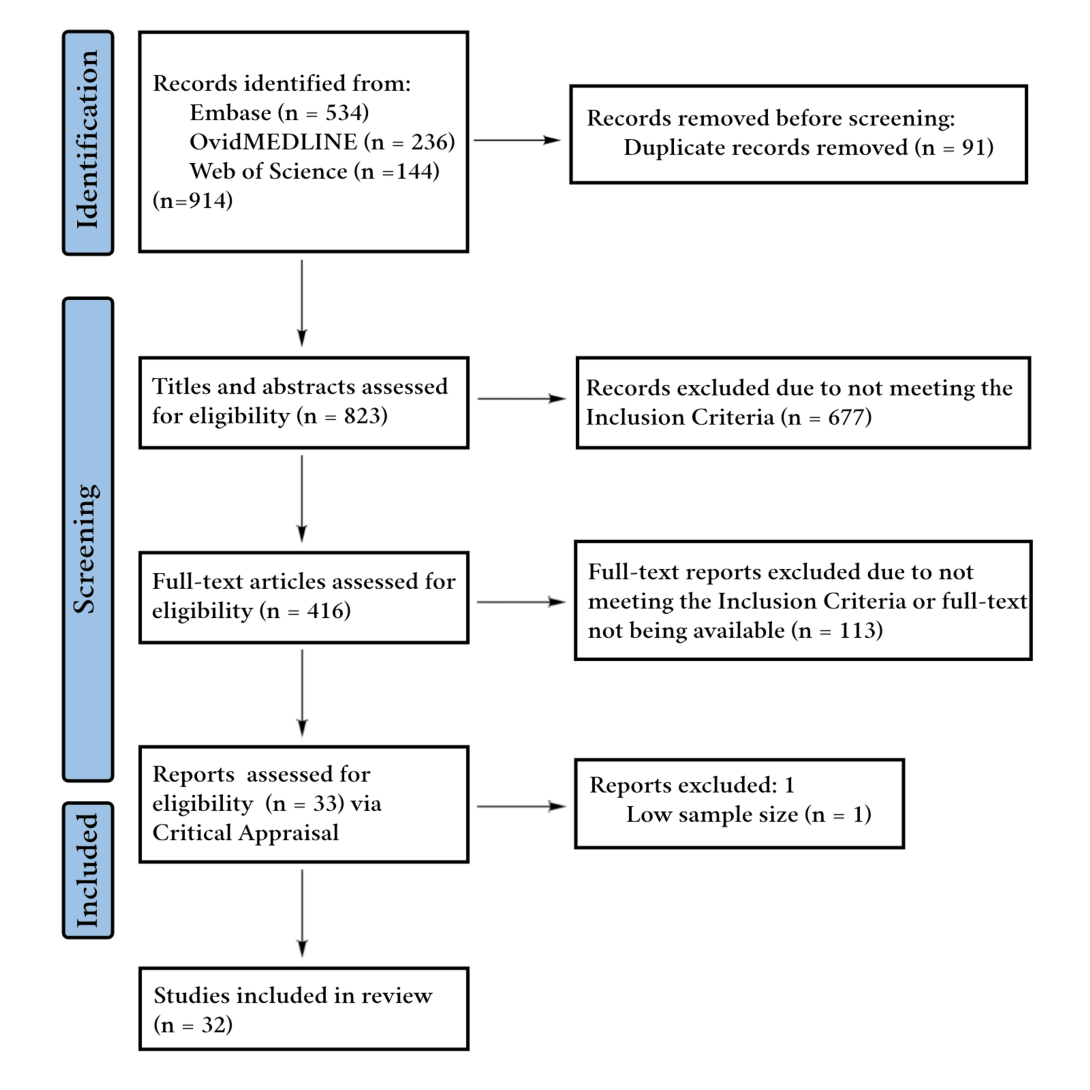
PRISMA diagram displaying the article screening process

Database searches identified 914 articles, of which 91 were duplicates and subsequently removed. Title/abstract screenings excluded 677 of the remaining 823 articles due to irrelevance to DM management or did not align with our inclusion criteria and were excluded, e.g., due to a different study population or incorrect publication types and/or irrelevant outcome(s). This left 146 articles for full-text screening. Of these, 113 were excluded during full-text screening for reasons such as unavailability of the full text, wrong study population, publication type, and/or outcome(s). Thirty-three articles were assessed for risk of bias, and one was excluded due to a small sample size (n = 1) and a high risk of bias. Of the remaining 32 articles, 31 had a low risk of bias (n = 16 had scores of 100%), and one had a moderate risk of bias. Ultimately, these 32 articles were included in the review. The article selection process is depicted in Figure 1. A detailed summary of each included article is provided in Table 4.

**Table 4:** Summary of the included articles BGM: blood glucose monitoring, BMI: body mass index, CGM: continuous glucose monitoring, COVID-19: coronavirus 2019, DKA: diabetic ketoacidosis, DM: diabetes mellitus, ED: emergency department, GMI: glucose management indicator, HbA1c: hemoglobin A1c, IQR: interquartile range, T1DM: type 1 diabetes mellitus, T2DM: type 2 diabetes mellitus, TIR: time in range.

Author(s)/year	Appraisal score	Methods and participants	DM measure(s)	Key finding(s)
Abdulhussein et al. [43]	100%	Retrospective cohort study. Data was collected from electronic health records, Dexcom Clarity (DexCom Inc., San Diego, CA, United States), and Tidepool. n = 85 youths, adolescents, and young adults with T1DM aged 3-21 years; mean age = 13.6 ± 4.6 years.	CGM metrics, HbA1c, insulin dosing, insulin delivery method, and carbohydrate consumption.	CGM metrics in youth with T1DM improved during the lockdown. In patients using insulin pumps, there were no significant differences in insulin doses or carbohydrate consumption before and during the lockdown.
Alguwaihes et al. [44]	75%	Cross-sectional study. Data was collected via survey. n = 1,010 patients with T1DM including n = 518 youths and adolescents aged ≤20 years.	HbA1c, frequency of glucose monitoring, severe hypoglycemia episodes, DKA episodes, communication with a healthcare provider, prior visits with healthcare provider, and access to insulin and diabetes supplies.	Patients who communicated with their physicians during the pandemic had a lower occurrence of hypoglycemia and DKA than those who did not communicate. Patients under 14 years of age had the highest rates of severe hypoglycemia. The frequency of BGM was not affected in most patients. More than 40% reported difficulties in getting insulin and diabetes supplies.
Bharill et al. [45]	100%	Retrospective study. Data was collected from electronic health records and EMR and the Care Everywhere database. n = 63 youths with T2DM under 21 years of age ; median age = 15 years; IQR = 14-16 years.	Number of emergency room visits and hospital admissions, number of endocrine clinic visits and missed visits, HbA1c, weight, and BMI.	There was no significant difference in weight or BMI before and during the COVID-19 pandemic; however, HbA1c significantly increased during the pandemic. There was no change in the mean number of endocrine visits, missed endocrine visits, emergency department (ED) visits, or hospitalizations before and during the pandemic. Before the pandemic, all clinic visits were in-person; during the pandemic, more than 62% were via telemedicine.
Brener et al. [46]	100%	Observational study. Data was collected from medical files and Dexcom Clarity. n = 102 youths and adolescents with T1DM aged 3-18 years; mean age = 11.2 ± 3.8 years.	CGM metrics, HbA1c, and insulin delivery method.	Glucose TIR improved among adolescents during the lockdown.
Calcaterra et al. [47]	81.8%	Observational study. Data was collected via interviews with parents/guardians of adolescents with T1DM through an online platform. n = 280 youths and adolescents with T1DM under 18 years of age; mean age = 11.8 ± 3.3 years.	BMI, HbA1c, metabolic compensation, CGM metrics, insulin delivery method, insulin dosing, physical activity level, and weight.	Adolescents’ physical activity levels decreased, while their mean glycemic values and insulin doses increased. An online exercise training program provided adolescents with moderately intense physical activities.
Cheng et al. [48]	75%	Cross-sectional study. Data was collected via interviews, questionnaires, and electronic health records. n = 93 youths and adolescents with T1DM (mean age = 11.1 ± 3.5 years) and 30 youths and adolescents with T2DM (mean age = 13.8 ± 2 years).	HbA1c, insulin delivery method, insulin dosing, glucose monitoring method, weight, BMI, physical activity level, screen time, dietary habits, and sleep habits.	Males, patients with T2DM, and pubertal adolescents had significant increases in HbA1c. Prepubertal patients with T1DM had improved HbA1c levels. Weight and BMI increased in T1DM patients but decreased in T2DM patients. Reduced meal frequencies and physical activities and increased screen time and sleep duration were observed in both groups.
Chobot et al. [49]	90.9%	Controlled study. Data was collected from the Better Control in Pediatric and Adolescent DiabeteS: Working to CrEate CEnTers of Reference (SWEET) database. n = 42,798 youths with T1DM aged ≤21 years; median age = 14.8 years; IQR = 11.2-17.7 years.	HbA1c, use of insulin pump, use of CGM, prevalence of DKA, severe hypoglycemia, BMI, insulin dosing, psychological support, and use of telemedicine.	Patients at healthcare centers that transitioned partially to telemedicine showed a steady increase in HbA1c between 2018 and 2021; those at centers that transitioned mainly to telemedicine improved HbA1c from 2018 to 2021. Insulin pump and CGM use significantly increased; however, the number of data uploads per patient decreased during the pandemic. Pre-COVID differences in HbA1c, DKA, and severe hypoglycemia between centers offering telemedicine and those new to telemedicine in 2018 decreased two years after the pandemic began. Additional psychological care was associated with lower HbA1c and fewer DKA and severe hypoglycemia episodes.
Choudhary et al. [50]	100%	Controlled, retrospective study. Data was collected through electronic health records and questionnaires. n = 1,600 youths and adolescents with T1DM.	HbA1c, CGM usage, CGM metrics, hospitalization frequency, depression, and medical visits.	Overall hospitalization frequency, glycemic control, and depression did not significantly change during the pandemic. Older age, noncommercial insurance, Black and Hispanic race/ethnicity, and non-utilization of CGM were associated with higher HbA1c levels.
Danne et al. [51]	100%	Cohort, controlled study. Data was collected from the SWEET database. n = 16,735 patients with T1DM aged ≤21 years in 2019 and n = 12,157 patients with T1DM aged ≤21 years in 2020.	HbA1c, CGM usage, insulin dosing, BMI, frequency of severe hypoglycemia, and DKA episodes.	HbA1c and rates of severe hypoglycemia remained mostly unchanged from the previous year. Rates of DKA increased significantly in countries with the highest COVID-19 mortality rate, before dropping to baseline after the wave. Use of CGM slightly decreased and then increased significantly again.
Derkaoui et al. [52]	76.9%	Randomized controlled trial. Data was collected from clinic visits and questionnaires. n = 62 patients with T1DM with a mean age of 15 ± 6.4 years.	HbA1c, number of hypoglycemia episodes, diabetes treatment satisfaction, insulin delivery method, and use of smartphone app.	Patients using a smartphone application for therapeutic education and insulin dose management had lower HbA1c levels and fewer hypoglycemic episodes per week. Diabetes treatment satisfaction was higher among smartphone application users than non-users.
Dovc et al. [53]	100%	Prospective observational study and retrospective analysis. Data was collected from previous visits, CGM reports, and video visits. n = 313 youths, adolescents, and young adults with T1DM under 23 years of age.	Insulin delivery method, glucose monitoring method, HbA1c, mean glucose concentration, number of glucose measurements, insulin dosing, carbohydrate consumption, DKA episodes requiring hospitalization, and severe hypoglycemia episodes requiring hospitalization.	During the lockdown, adolescents receiving care via telemedicine had slight improvements in glycemic control. Total daily dose of insulin and carbohydrate consumption increased during the lockdown. There were no severe hypoglycemic or DKA episodes or hospitalizations during the period of study.
Elhenawy and Eltonbary [54]	62.5%	Cross-sectional study. Data was collected via questionnaires. n = 115 youths and adolescents with T1DM under 18 years of age.	HbA1c, insulin dosing, insulin delivery method, glucose monitoring method, carbohydrate counting, frequency of hypoglycemia and hyperglycemia, frequency of glucose monitoring, dietary habits, physical activity level, method of communication with a healthcare provider, satisfaction with communication with a healthcare provider, stress, and worry regarding access to supplies.	More than 60% of patients had worse HbA1c levels after the lockdown, and nearly all were able to continue follow-up through telemedicine. Nearly 60% of patients had worse eating habits. Hyperglycemia and hypoglycemia were more frequent during the lockdown. Only 5% exercised at home during the lockdown. Most reported moderate stress; stress was positively associated with HbA1c before and after the lockdown. Fear of hospital admission and shortage of medical supplies were the main worries.
Hakonen et al. [55]	100%	Retrospective study. Data was collected from the registry before and during the lockdown. n = 245 youths and adolescents with T1DM aged 4-16 years; mean age = 11 years.	CGM metrics, insulin delivery method, insulin dosing, insulin pump type, CGM type, and HbA1c.	Overall glycemic control was similar before and during the lockdown. Adolescent females using insulin pumps had significantly improved glycemic control during the lockdown. Total daily insulin doses did not change significantly before and during the lockdown.
Hammersen et al. [56]	100%	Controlled, cohort study. Data was collected from the Diabetes Prospective Follow-Up (DPV) registry. n = 19,729 youths and adolescents with T1DM under 18 years of age; median age = 12.6 years.	Insulin dosing, use of insulin pump, use of CGM, CGM metrics, BMI, weight, HbA1c, DKA episodes, number of hospitalizations, and length of hospitalizations.	There was no significant difference in metabolic control before, during, or after the lockdown. CGM metrics, insulin doses, and BMI were slightly higher. Patients using CGM in 2019 and 2020 had improved CGM metrics.
Hammersen et al. [57]	100%	Observational study. Data was collected from the DPV registry. n = 33,372 youths and adolescents with T1DM under 18 years of age; median age = 14.3 years.	BMI, weight, HbA1c, CGM metrics, hypoglycemia episodes, and DKA episodes.	There was no significant change in glycemic control or acute complications during the pandemic. BMI and insulin doses increased during the pandemic.
Hasan Tehrani et al. [58]	87.5%	Cross-sectional study. Data was collected via interviews with parents and youths and electronic health records. n = 98 youths and adolescents with T1DM; mean age = 13.5 ± 4 years.	HbA1c, mean fasting and postprandial blood glucose, weight, BMI, insulin dosing, number of hypoglycemic events, number of DKA episodes, availability of insulin, access to medical services or health facilities, sleep habits, number of blood glucose measurements, physical activity levels, and hospitalizations.	There was a significant change in sleep habits, insulin doses, daily exercise, and availability of insulin before and during the outbreak. The frequencies of hospitalizations and hypoglycemic events were lower after the COVID-19 outbreak. There was no significant difference in HbA1c before and after the outbreak.
Kaushal et al. [59]	100%	Controlled, retrospective study. Data was collected via electronic health records. n = 555 youths and adolescents with T1DM aged 1-17 years; mean age = 12.3 ± 3.4 years.	CGM metrics, HbA1c, and frequency of medical visits.	Diabetes visit frequency increased from pre-pandemic to during the pandemic. During the pandemic, 92% of visits were virtual. Glucose management indicator (GMI) improved slightly from pre-pandemic to during the pandemic. Patients with equal or greater visit frequencies had significant improvements in GMI, whereas those with lower visit frequencies did not.
Kaushal et al. [60]	100%	Retrospective, controlled study. Data was collected from electronic health records. n = 641 youths and adolescents with T1DM aged 1-17 years pre-pandemic (mean age = 12.3 ± 3.5 years) and n = 648 youths and adolescents with T1DM aged 1-17 years during the pandemic (mean age = 13.3 ± 3.5 years).	HbA1c, CGM metrics, and use of an insulin pump, CGM, and telemedicine.	CGM metrics were significantly improved during the pandemic compared to pre-pandemic. More patients achieved GMI < 7% during the pandemic than the prior year. Before the pandemic, 0.1% of visits were virtual; during the pandemic, 93.5% of visits were virtual.
Lazzeroni et al. [61]	90.9%	Observational, retrospective study. Data was collected from medical records and interviews. n = 139 youths and adolescents with T1DM aged 2-20 years; mean age = 13.9 years.	HbA1c, BMI, glucose monitoring method, insulin delivery method, insulin dosing, type of clinic visits, frequency of contact with clinic, diet modifications, frequency of meals with family, and physical activity level.	There was a significant decrease in HbA1c, especially among patients with previously unsatisfactory glycemic control. During the lockdown, there were significantly more telemedicine contacts. Physical activity levels significantly decreased; however, BMI did not increase. Most patients reported changing their diet habits, including more snacks, skipping meals, and sharing meals with family members.
Miller et al. [62]	90.9%	Observational study. Data was collected via surveys completed by parents and youths prior to and during the pandemic. n = 43 adolescents with T1DM aged 10-19 years (mean age = 15.3 ± 2.2 years) and n = 88 parents.	Emotional regulation, stress, anxiety, diabetes distress, usage of CGM, insulin delivery method, and family involvement in diabetes management.	At the start of the pandemic, the parents’ responsibility for youth T1D management increased, and their diabetes-related stress decreased. Decreased emotional functioning over time was related to family pandemic-related stress. Youth self-regulation, specifically behavioral and emotional aspects, predicted better emotional and T1DM functioning.
Neo et al. [63]	80%	Qualitative, cross-sectional, cohort study. Data was collected via surveys completed by youths, adolescents, and parents of youths with T1DM. n = 92 youths and adolescents with T1DM aged 5-16 years.	HbA1c, insulin delivery method, satisfaction with diabetes management, dietary habits, physical activity level, depression, anxiety, and telehealth vs. in-person appointments.	Adolescents who reported feeling more depressed and alone had a higher HbA1c. HbA1c was significantly inversely correlated with parents’ perceptions of T1DM treatment goals. Adolescents had decreased physical activity levels and made changes in diet and insulin requirements. Parents’ perceptions of diet and insulin changes during the lockdown were significantly associated with HbA1c.
Nwosu et al. [64]	100%	Observational study. Data was collected via electronic health database 1-4 months before the lockdown and 1-4 months after the lockdown. n = 110 pediatric patients with T1DM with a mean age = 14.8 ± 4.9 years.	CGM usage, CGM metrics, insulin pump usage, insulin dosing, HbA1c, BMI, weight, lipids, cholesterol level, and triglycerides.	No change in HbA1c was noted during the lockdown. Significant predictors of lower HbA1c levels were pre-lockdown HbA1c and CGM usage. Non-CGM users had significantly increased total daily insulin doses but no change in HbA1c.
O’Donnell et al. [65]	90%	Qualitative, controlled study. Data was collected via self-reported surveys. n = 122 adolescents with T1DM aged 13-18 years.	HbA1c, blood glucose variability, CGM usage, insulin pump usage, diabetes burden and distress, eating habits, physical activity level, diabetes supplies, family involvement in diabetes management, access to the healthcare team, and use of telehealth.	Adolescents reported the pandemic affected their T1DM management; the most common areas were food, self-care, health/safety, diabetes appointments, and exercise. Compared to adolescents who reported minimal difficulty managing T1DM during the pandemic, those reporting moderate-to-extreme difficulty were more likely to have HbA1c levels ≥ 8%. Most reported continued access to their healthcare team during the pandemic, and they were not arguing more with their parents regarding T1DM management.
Santana and Del Roio Liberatore [66]	87.5%	Cross-sectional, observational study. Data was collected via teleconsultations. n = 143 youths, adolescents, and young adults with T1DM aged 0-20 years.	HbA1c, number of medical phone calls, insulin dosing, and hospitalizations due to diabetes decompensation.	Teleconsultation promoted healthcare for patients with DM; however, it did not guarantee sufficient glycemic control. HbA1c improved in 37% of patients but worsened in 43%. Nearly 85% of patients needed to change insulin doses, and a few hospitalizations occurred due to decompensation.
von Sengbusch et al. [67]	100%	Observational, cohort study. Data was collected via phone or video contacts. n = 100 youths and adolescents with T1DM aged 3-18 years; mean age = 11 ± 4.1 years.	HbA1c, CGM metrics, severe hypoglycemia episodes, hospitalizations, and use of telehealth.	HbA1c levels remained stable, and there was a slight improvement in CGM metrics among patients receiving care via telemedicine.
Shah et al. [68]	100%	Observational study. Data was collected via medical and electronic records. n = 312 youths, adolescents, and young adults with T1DM aged 2-21 years.	BMI, HbA1c, physical activity level, waist circumference, body fat percentage, and lipid parameters.	Glycemic control improved during COVID-19 restrictions compared to pre-COVID-19 restrictions. Physical activity level was significantly lower. Waist circumference, body fat percentage, and lipid parameters increased. There was no significant change in BMI.
Smudja et al. [69]	87.5%	Cross-sectional study. Data was collected via questionnaires, patient diaries, and electronic health records. n = 182 youths and adolescents with T1DM.	HbA1c, insulin delivery method, usage of CGM, DKA, severe hypoglycemia, presence of comorbidities, and quality of life.	The main determinants of quality of life were glycemic control protocol adherence, presence of comorbidities, level of metabolic control, and type of insulin therapy. Pediatric patients with comorbidities, poor metabolic control, and those receiving human insulin reported a significantly higher presence of pain/discomfort, anxiety/depression, or suboptimal assessment of health status. Fifty percent did not adhere to therapeutic and dietary guidelines agreed upon with their physicians. Older age was associated with better social and peer support; however, those aged 13–18 years had the lowest subjective health status rating.
Telford et al. [70]	75%	Cross-sectional study. Data was collected via self- and proxy-reported questionnaires and electronic health records. n = 33 adolescents with T1DM aged 11-18 years (mean age = 14.1 ± 1.6 years), and a control group of n = 34 adolescents without T1DM aged 11-18 years (mean age = 14.6 ± 1.9 years).	Physical activity level, BMI, weight, insulin delivery method, blood glucose monitoring method, HbA1c, severe hypoglycemia, and hospital admissions with DKA.	Both adolescents with T1DM and a control group did not receive recommended levels of physical activity during the lockdown. Younger adolescents were more active than older adolescents. Physical activity level was negatively associated with BMI. Having T1DM predicted an increase in BMI. There was a moderate-to-strong positive correlation between HbA1c and BMI.
Tinti et al. [71]	90.9%	Cohort study. Data was collected via remote visits 90 days before and 90 days during the lockdown. n = 66 youths and adolescents with T1DM aged 0-18 years; mean age = 11.6 ± 4.5 years.	CGM metrics, HbA1c, insulin dosing, and physical activity level.	Patients’ physical activity levels decreased during the lockdown, likely leading to a higher total daily insulin dose. Patients had slight improvements in CGM metrics. There were no significant differences in CGM metrics or total daily insulin dose in patients who received remote care during the lockdown.
Turan et al. [72]	100%	Controlled, cross-sectional study. Data was collected from outpatient visits 30 days before the quarantine and 15 days after it was over. n = 100 youths and adolescents with T1DM under 18 years of age; mean age = 14.7 ± 3.4 years.	Snack and meal frequency, carbohydrate consumption, HbA1c, exercise pattern, BMI, weight, insulin dosing, number of hypoglycemic episodes, and sleep habits.	The mean HbA1c level was significantly higher after the lockdown compared to before. Meal schedules changed due to delayed sleep and waking times, and total daily carbohydrate consumption increased in the subgroup with increased HbA1c while it decreased in the subgroup with decreased HbA1c. Physical activity levels decreased in both subgroups during the lockdown.
Zeiler et al. [73]	90%	Qualitative study. Data was collected via interviews with adolescents and their parents and clinic visits. n = 10 adolescents with T1DM aged 15-18 years and n = 8 parents.	Experiences during confinement regarding everyday life, eating habits, physical activity level, well-being, HbA1c, BMI, insulin delivery method, glucose monitoring method, insulin dosing, stress, adaptation to changed routines, access to insulin and supplies, need for medical support, telehealth usage, and self-responsibility/family involvement in diabetes management.	Digital diabetes treatment was well-accepted and viewed as highly usable. The pandemic either aided or hindered the transition from parental control to diabetes self-management. Some patients were able to improve healthy lifestyle behaviors, while others found it difficult to adapt, resulting in more sedentary behavior and snacking. Perceived stress was associated with blood glucose levels.
Zubkiewicz-Kucharska et al. [74]	100%	Retrospective, cohort study. Data was collected from at least two in-person visits in 2020, as well as in 2021. n = 185 youths and adolescents with T1DM aged 2-18 years; mean age = 11.5 ± 3.5 years.	BMI, weight, CGM metrics, HbA1c, and insulin dosing.	During the first few months of the study period, HbA1c levels improved. BMI and total daily insulin doses increased significantly; however, BMI returned to baseline later.

Study Characteristics

The 32 included articles presented data from 19 countries, including the United States (n = 8) [43,45,50,59,60,62,64,65], Germany [56,57,67] and Italy [47,61,71] (n = 3 each), and Australia [63], Austria [73], Brazil [66], Egypt [54], Finland [55], India [68], Iran [58], Israel [46], Malaysia [48], Morocco [52], New Zealand [70], Poland [74], Saudi Arabia [44], Serbia [69], Slovenia [53], and Turkey [72] (n = 1 each). Two articles presented data from multiple countries [49,51]. The majority of the studies (n = 30) focused solely on participants with T1DM, while one study only included participants with T2DM [45], and another included participants with both T1DM and T2DM [48]. The number of participants per study varied widely, ranging from a qualitative interview study [73] with 10 participants to an observational registry study with n = 42,798 [49].

Across the articles, the most frequently reported measures of adolescents’ T1DM and T2DM self-management during the COVID-19 pandemic included glycemic control and monitoring frequency (e.g., HbA1c values and CGM metrics; n = 32 articles), followed by medication/insulin administration and regimens (n = 26 articles) [43,44,46-49,51-56,58,60-66,69-74], weight management and lifestyle behaviors (e.g., BMI values, nutrition habits, and physical activity levels; n = 22 articles) [43,45,47-49,51,53,54,56-58,61,63-65,68-74], hospitalizations and acute diabetes complications (e.g., hypoglycemia and DKA; n = 16 articles) [44,45,49-54,56-58,66,67,69,70,72], and engagement in outpatient care (e.g., attendance at in-person or virtual visits, medical support apps, and communication with healthcare providers; n = 15 articles) [44,45,49,50,52,54,58-61,63,65-67,73]. Ten articles also addressed psychosocial indicators of adolescents’ diabetes self-management (e.g., depression, stress, satisfaction with treatment, and familial support) [49,50,52,54,61-63,65,69,73]. 

Glycemic Control and Monitoring

Studies included in our scoping review reported varied results pertaining to glycemic management among adolescents with T1DM and T2DM during the COVID-19 pandemic. Eight articles noted that adolescents’ glycemic control remained stable during the pandemic [50,51,55-58,64,67]. Adolescents who regularly used CGM devices and insulin pumps, made appropriate insulin dosing adjustments, reported lower stress, and received healthcare and support via telemedicine or smartphone apps typically had stable or improved glycemic control during the pandemic [43,46,49,50,52-56,59-65,67,70-73]. A study including approximately 40,000 participants with T1DM found that their median HbA1c value significantly decreased by 0.2% (1 mmol/mol) between 2018 and 2021; inversely, their utilization of insulin pumps and CGMs significantly increased by 7.8% and 22.3%, respectively, during this time period [49]. Another study including more than 20,000 participants with T1DM found that their aggregate HbA1c values between 2019 and 2020 were comparable; however, their CGM usage changed significantly: first by decreasing early in the pandemic (53% vs. 51%) and later increasing (55% vs. 63%) [51]. Studies also indicated that adolescents who engaged less frequently in BGM, did not use CGM devices or insulin pumps, reported higher stress, did not follow healthy lifestyles, and had lower utilization of telemedicine typically had worse glycemic control during this time period [43,46,49,50,52-56,59-65,67,69-73]. One study noted that glycemic outcomes were significantly worse among non-Hispanic Black and Hispanic adolescents, highlighting racial/ethnic disparities [50].

Medication Regimens, Weight Management, and Lifestyle Behaviors

Thirteen studies reported that adolescents with T1DM increased their insulin doses and gained weight during the COVID-19 pandemic [47,48,53,54,56-58,63,64,66,70,71,74]. Interestingly, a study comparing outcomes between adolescents with T1DM and T2DM found that those with T1DM gained weight, whereas those with T2DM lost weight [48]. Another study found having T1DM predicted BMI increase, when comparing outcomes between adolescents with and without T1DM [70]. A study of over 33,000 participants with T1DM found that their BMI-standard deviation score (BMI-SDS) increased from 0.29 (95% confidence interval = 0.28-0.30) in late 2019 to 0.40 (95% confidence interval = 0.39-0.41) in late 2021 [57]. Changes in adolescents’ weight and insulin doses were often attributed to increased carbohydrate consumption during their extended amount of time spent at home [53,54,61,63,65,68,69,72,73]. Furthermore, most studies examining physical activity levels among adolescents with DM during the pandemic found that this age group received significantly lower levels of physical activity while under lockdown measures [47,48,54,58,61,63,65,68,70-73]. One study noted that none of the enrolled participants, including those with and without T1DM, met the recommended physical activity levels during this time period [70]. The same study stratified physical activity levels by age and found that younger adolescents received more physical activities than older adolescents during the pandemic [70]. Three studies noted that adolescents also modified their sleep habits and engaged in more screen time during lockdown measures, which likely exacerbated their metabolic changes [48,58,72].

Inpatient Care, Acute Diabetes Complications, and Psychosocial Issues

Studies included in our review presented varying results pertaining to rates of acute complications, such as DKA and severe hypoglycemia, as well as hospitalizations among adolescents with DM during the pandemic. Acute complications occurred more frequently among adolescents who did not utilize telemedicine services, did not maintain regular communication with their healthcare providers, and/or contracted COVID-19 [44,45,49-54,56-58,66,72]. One study found that adolescents under 14 years of age had more frequent episodes of severe hypoglycemia compared to other age groups [44]. Another cross-national study reported that rates of DKA increased significantly among adolescents with T1DM residing in countries with the highest COVID-19 mortality rate; however, these rates decreased later [51].

Six studies identified mental health issues affecting adolescents with DM throughout the COVID-19 pandemic, such as depression, isolation, and stress [49,54,62,63,65,73]. As noted previously, these issues were negatively associated with adolescents’ glycemic and metabolic outcomes. Furthermore, parents/guardians’ level of involvement in adolescents’ DM management influenced the adolescents’ glycemic and metabolic outcomes [61,62,65,73]. One study indicated that the pandemic either facilitated or hindered transitions in parents/guardians’ oversight of their adolescents’ DM management, affecting the adolescent patients’ sense of independence over their own self-management [73]. 

Outpatient Care and Telemedicine Utilization

Lastly, 13 studies found that telemedicine and other remote-delivered diabetes care services were widely utilized and viewed positively by adolescents with DM during the COVID-19 pandemic [44,45,49,50,52,54,59-61,65-67,73]. Utilization of additional mental health services during the pandemic was significantly associated with lower HbA1c values and fewer episodes of DKA and severe hypoglycemia in one study of ~40,000 participants with T1DM [49]. Some adolescents with T1DM also utilized mobile apps designed to provide diabetes education during the pandemic; a small randomized controlled trial found a therapeutic education and insulin dosing app to be associated with a significant decrease in adolescents’ frequency of hypoglycemia episodes and a significant increase in their treatment satisfaction [52]. As noted above, most studies found that adolescents who utilized telemedicine and remote-delivered healthcare services were more likely to maintain or improve their glycemic management and were less likely to be hospitalized and experience acute complications.

Discussion

With the increasing global prevalence of T1DM and T2DM among adolescents, the management of these conditions among this age group constitutes a major public health concern [7]. Previous research emphasizes that people with T1DM and T2DM face a multitude of barriers to self-management adherence, including a lack of understanding regarding diabetes care and dietary plans, communication challenges with their families and healthcare providers, and limited socioeconomic resources [20]. The COVID-19 pandemic introduced additional barriers stemming from lockdowns that restricted adolescents’ access to diabetes care, school-based nutrition and physical activity programs, and social support [32,33].

Our scoping review elucidates the impacts of the COVID-19 pandemic on adolescents’ management of T1DM and T2DM, globally [43-74]. The articles included in our scoping review had varied findings; however, we identified several recurring themes in their results. First, numerous studies found that adolescents’ overall glycemic control remained comparable from before the pandemic to during the pandemic [50,51,55,57,58,64,67,71]. However, several studies noted that some adolescents experienced an improvement in glycemic control while others did not during this time period [43,46,49,50,52-56,59-65,67,69-73]. In these cases, adolescents’ glycemic changes were primarily attributed to their usage/lack of usage of CGM, adjustments in insulin dosing, physical activity, stress level, and utilization or lack of remote-delivered care [43,46-48,53,54,58,61,63,65,68-73]. The studies also reported varying results regarding rates of hospitalizations, DKA, and hypoglycemia among adolescents with T1DM or T2DM during the COVID-19 pandemic [44,45,49-54,56-58,66,72]. These variations align with differences in adolescents’ overall glycemic control during this time period.

Most studies examining adolescents’ weight and lifestyle behaviors reported that these patients gained weight and had higher BMIs during the pandemic [47,48,53,54,56-58,63,64,66,70,71,74]. These metabolic changes were attributed to adolescents receiving lower levels of physical activity due to COVID-19 restrictions. Adolescents’ increased amount of time spent at home resulted in having more sedentary lifestyles. They also engaged in more frequent snacking, consumed more carbohydrates, had increased screen time, and changed their sleep schedules. Several studies noted that adolescents and their parents/guardians reported psychosocial issues during the pandemic, which also negatively affected their overall health [49,54,61-63,65,73]. These issues included elevated stress levels, worries about access to insulin and diabetes supplies, and transitions in DM management between adolescents and their parents/guardians.

Moreover, most studies examining outpatient care among adolescents with T1DM and T2DM during the pandemic reported these patients had high utilization of telemedicine, which helped them maintain glycemic control [44,45,49,50,52,54,59-61,65,67,73]. Telemedicine and remote communication with diabetes care providers enabled adolescents and their parents/guardians to receive guidance about insulin dosing decisions, lifestyle changes, and other strategies to manage their condition more efficiently while at home. Telemedicine also enabled them to receive consistent follow-up care while simultaneously mitigating their risk of COVID-19 exposure from in-person clinic visits.

Although most articles included in our scoping review did not identify significant changes in adolescents’ glycemic control during the COVID-19 pandemic, it is clear these patients experienced a multitude of health, behavioral, and psychosocial issues. The long-term ramifications of this public health crisis for this age group and patient population are still not fully known. As such, our review demonstrates the need for further research and interventions aimed at ensuring adolescents with T1DM and T2DM receive necessary medical care and support over the long term. In particular, innovative research and interventions aimed at improving adolescents’ lifestyle habits and mental health are urgently needed, as this review indicates behavioral and psychosocial issues negatively affect their glycemic management. On the contrary, since this review found that adolescents’ utilization of CGM, insulin pumps, and telemedicine helped them to maintain or improve their glycemic control, interventions focused on empowering more adolescents to use these medical devices and services could be highly valuable. Furthermore, research and policy changes aimed at expanding access to these devices and remote-delivered healthcare services among underserved patients, such as racial/ethnic minorities, those living in rural or under-resourced areas, and those with lower socioeconomic status are urgently needed. Ensuring the representation of these vulnerable patients in future DM and COVID-19 studies is critical, given their worse outcomes [31]. Lastly, our scoping review provides a foundation for reflection on challenges associated with pediatric diabetes care and research during the COVID-19 pandemic and opportunities to prepare for potential unforeseen crises in the future, including another pandemic.

Few limitations exist in the methodology of this scoping review. We followed the PRISMA-ScR checklist and employed a rigorous article screening process to reduce bias, including a calibration exercise, IRR calculation, and critical appraisals, for which nearly all included articles were determined to have a low risk of bias. Despite these efforts, there is a possibility that bias still could have occurred in the article selection process. Limitations also exist within the research articles selected for our scoping review. As the COVID-19 pandemic was unprecedented and healthcare delivery and research efforts rapidly changed during this time period, some researchers abruptly changed their data collection methods or were unable to properly measure certain health indicators [75]. For example, telemedicine affected researchers’ ability to obtain certain lab values (e.g., Hb1Ac), or these values were measured at inconsistent time intervals. Numerous studies utilized patients’ and parents/guardians’ self-reported data from surveys and interviews, which have the potential for various self-selection and self-report biases [76,77]. Additionally, numerous studies included participants outside the adolescent age range of 10-17 years; therefore, certain data points from these studies were aggregated from a larger age group. Lastly, the included studies employed a mix of longitudinal and cross-sectional research designs, which posed challenges for comparing their results.

This review stands out as the first, to our knowledge, to examine the impact of COVID-19 on glycemic management, lifestyle habits, and psychosocial well-being for adolescents with T1DM and T2DM. Our review’s comprehensive analysis of 32 articles presenting data from 19 countries emphasizes the breadth of research in this area and underscores the global reach of COVID-19’s impact on adolescents with DM. This review not only informs clinical strategies for supporting adolescents with DM during pandemics but also has the potential to influence public health practices, fostering preparedness for similar disruptions in the future.

## Conclusions

This scoping review examined T1DM and T2DM self-management regimens and barriers to adherence among adolescents during the COVID-19 pandemic. Most studies noted that adolescents did not significantly worsen their glycemic control during the pandemic. Adolescents who regularly monitored their blood glucose levels, adjusted their insulin regimens, and received healthcare through telemedicine maintained glycemic stability or even improved glycemic control. Rates of acute complications such as DKA among adolescents varied during the pandemic. Numerous studies reported that adolescents had higher BMIs, reduced physical activity levels, higher carbohydrate consumption, increased daily insulin doses, and elevated stress during the pandemic. Further research and interventions are needed to improve adolescents’ DM outcomes, behaviors, and access to care. In particular, efforts aimed at empowering adolescents with T1DM and T2DM to increase their physical activity levels, maintain healthy eating habits, and reduce stress are needed.
