# Correction: Adolescents’ Diabetes Self-Management Regimens and Outcomes During the COVID-19 Pandemic: A Scoping Review 

**DOI:** 10.7759/cureus.c334

**Published:** 2025-10-01

**Authors:** Jason Nguyen, William Le, Roberta Brugger, Anjali Shah, Prasanna Karur, Macey Hedelund, John Joseph, Arshia Haj, Caroline Grillo, Nivene Hojeij, Jennifer Maizel

**Affiliations:** 1 Diabetes and Endocrinology, Nova Southeastern University Dr. Kiran C. Patel College of Osteopathic Medicine, Tampa, USA; 2 Diabetes and Endocrinology, Nova Southeastern University Dr. Kiran C. Patel College of Osteopathic Medicine, Fort Lauderdale, USA; 3 Public Health, Nova Southeastern University Dr. Kiran C. Patel College of Osteopathic Medicine, Fort Lauderdale, USA; 4 Behavioral Health and Health Policy Practice, Westat, Rockville, USA

This article has been corrected to fix the following typo in the PRISMA diagram: "Full-text articles assessed for eligibility" corrected from 416 to 146.

